# Beyond the curriculum: a comprehensive advising approach to strengthen MPH students’ professional readiness

**DOI:** 10.3389/fpubh.2026.1920861

**Published:** 2026-08-05

**Authors:** Jasmine Hamilton, Sharia Phillips, Twynette Mixon, Wayne McCullough

**Affiliations:** Charles Stewart Mott Department of Public Health, Michigan State University, Flint, MI, United States

**Keywords:** academic advising, collaborative approach, partnerships, professional readiness, student development pathways

## Abstract

Preparing the next generation of public health practitioners and leaders to enter the workforce with the knowledge, skills, and confidence needed for professional success is an increasingly complex challenge for schools and programs of public health. This challenge is compounded by a persistent gap between what graduates learn and how they perceive their professional readiness. Professional readiness is shaped by access to career development opportunities, experiential learning, and meaningful engagement with public health professionals, instructors, and advisors. Addressing this preparedness gap requires extending support beyond the formal curriculum and foundational competencies through which students develop and apply public health knowledge. A comprehensive advising approach can play a critical role in strengthening students’ professional self-efficacy and perceived readiness for the workforce. We propose an advising continuum that integrates both transactional and transformational advising practices, spanning prescriptive and collaborative approaches. Through intentional and overlapping touchpoints, advisors in particular can support students’ confidence in navigating the placement and engagement into the workforce. Findings from our advising survey indicate that students who engage in our comprehensive advising continuum have reported enhanced advising experiences and increased self-efficacy related to professional readiness and career preparation.

## 1 Introduction

Public health education is essential for preparing the next generation of practitioners and leaders. For Master of Public Health (MPH) students, that preparation derives from a competency-based curricula, forged from applied practice and measurable outcomes that align with professional standards (1). Typically, MPH students show competency attainment through classroom-based assessments during their course work which then culminates with an applied practice experience where students apply what they learn in a public health setting. This training ensures MPH students graduate with a strong foundation in public health competencies and professional standards; however, a persistent gap remains between what graduates learn in academic settings and how they perceive their readiness to apply those skills in the workforce. These concerns stem from curricular and non-curricular contributors like limited opportunities to applied experiences, networking opportunities, and career supports that undermine graduates’ professional self-efficacy (2). We know that experiential and practice-based learning internships, practical, community-engaged projects, and courses have shown consistent promise for narrowing the academic-to-practice divide. These approaches develop problem solving, communication, and contextual skills critical for public health practice (3, 4). However, curricular innovations and experiential placements alone will not always ensure graduates’ professional readiness self-efficacy.

We contend that advising can serve as another pathway to close the academic-to-practice divide, one that we have shaped into an advising continuum framed by the idea that the advising process is fluid and should include both transactional and transformational practices. This continuum coordinates advising and career-development supports derived from seeking internal and external partnerships to help students build professional identity, workplace skills, and networks creating multiple pathways toward degree completion, professional growth and career confidence.

Advising reconceptualized as a multi-dimensional, as an intentionally sequenced process can reinforce applied learning, link students to mentorship and placement opportunities, and support career self-efficacy. Contemporary advising scholarship emphasizes proactive, institutionally supported approaches (including intrusive/proactive advising) and the potential for advising transformation particularly when advisors are empowered with technologies, institutional practices, and partnerships (5, 6). In this paper, we present an advising-continuum model that integrates transactional and transformational advising practices across prescriptive, intrusive, holistic, and collaborative dimensions. We argue that intentional, overlapping advising touchpoints can strengthen students’ academic and professional growth to align with increasing their confidence related to professional readiness.

## 2 Background

### 2.1 Traditional paradigms and the pathway from transactional to transformational practices

Academic advising has long served as a cornerstone of student support in higher education, yet its traditional models have been shaped primarily by a prescriptive, information-delivery orientation focused on course selection, degree requirements, and academic progress monitoring (7, 8). Within this paradigm, advisors function largely as gatekeepers of institutional policy, ensuring students meet compliance with benchmarks and progress toward graduation. While this approach addresses essential administrative functions, it leaves largely unaddressed the developmental and professional dimensions of student growth that have become increasingly central to post-graduation impact. This prescriptive orientation shows a more transactional engagement and practice within advising.

Shifts in student expectations have increasingly challenged the sufficiency of traditional advising models. Today’s graduate students, and particularly those enrolled in degree programs like an MPH, arrive with explicit career goals and seek advising experiences that actively support their entry or advancement in their chosen fields (9). Students now expect advisors to serve not only as academic guides but as career development partners who can connect them to professional networks, skill-building opportunities, and field-relevant experiences. This shift reflects a broader cultural movement toward outcome-oriented education, where the value of a graduate degree is increasingly measured by graduates’ readiness to contribute meaningfully to the workforce upon completion. This evolution in student expectations signals an addition in advising practice to a more proactive outreach and early intervention, away from purely transactional, prescriptive interaction toward more intrusive and holistic approaches that proactively engage students and attend to their development as whole people and emerging professionals.

As student expectations have evolved, so too has the recognized scope of the advisor’s role. The literature reflects a growing consensus that effective advising at the graduate level extends well beyond course scheduling and compliance monitoring toward something more relational and individualized, what is labeled as holistic advising which has also been described as the human art of building genuine connections that help students link their personal strengths and interests to their broader academic and life goals (10). Holistic advising recognizes the student as a whole person: their academic progress, professional aspirations, personal circumstances, and long-term goals are all relevant to the advising relationship. This orientation is particularly consequential for graduate students who, having already made a significant investment in their education, often require an advisor who knows them personally, advocates on their behalf, and engages them beyond the boundaries of compliance monitoring. Scholars in the field have increasingly called for advisors to function as student advocates: facilitating access to high-impact practices, fostering meaningful connections with faculty and professionals, and sustaining their engagement beyond the boundaries of the formal advising relationship (11, 12). This holistic orientation, representing what the literature describes as transformational advising: the relational, developmental interactions that extend beyond compliance toward growth, building students’ confidence, professional identity, and self-efficacy as emerging practitioners (13) is especially relevant for online MPH students who may exit their programs without the informal institutional ties that in-person settings naturally cultivate (14).

Taken together, prescriptive, intrusive, and holistic advising represent distinct but overlapping orientations, each moving student progressively from transactional compliance toward transformational development. While each approach addresses a different dimension of student need, no single orientation alone is sufficient to fully prepare graduate students for the demands of the public health workforce. These workforce demands have been documented through employer surveys, CEPH accreditation criteria, and national workforce studies consistently identify gaps between academic preparation and the applied, interpersonal, and adaptive competencies the field requires of new graduates, including leadership, systems things, and professional identity (1, 15–17).

### 2.2 Advising as a pathway to increase students’ perceived professional readiness

In public health specifically, professional readiness has taken on renewed urgency considering evolving employer expectations and the complex challenges facing the field. Professional readiness, defined as the attainment and demonstration of requisite competencies, confidence, and self-efficacy that broadly prepare graduates for a successful transition into the workplace (13, 17), has become a central outcome expectation for MPH programs, yet one that competency-based curricula alone have struggled to fully deliver. Skills Gap Theory helps explain this persistent tension: the gap between what education produces and what employers need is structural, reflecting a fundamental misalignment between academic preparation and the applied, interpersonal, and adaptive skills demanded in real-world practice settings (15). The need for MPH programs to actively adapt to evolving workforce expectations has been well documented, particularly around competencies such as leadership, systems thinking, and innovation, areas that CEPH accreditation standards require but that programs often address inconsistently and with limited opportunity for applied reinforcement (1, 16). Taken together, these pressures reveal the limits of treating professional readiness as a curricular outcome alone and point toward the need for complementary mechanisms, such as intentional advising and mentoring, that can bridge the gap between what students learn in the classroom and what the field requires of them upon entry.

Beyond technical competencies, public health employers increasingly expect graduates to demonstrate professional identity, career readiness, and the capacity to work effectively across interdisciplinary and community-based settings (18, 19). This includes having developed professional networks, the ability to articulate career goals, and confidence in navigating the job market. Yet the literature consistently identifies a gap between what MPH programs emphasize academically and what students need to successfully transition into the workforce, a structural mismatch that is particularly pronounced in fully online programs, where the conditions of remote learning, physical separation from campus resources, and reduced informal interaction with peers and faculty compound the challenge of professional development and self-efficacy building (20). Despite the growing emphasis on workforce preparation within public health, the integration of advising and mentoring as deliberate vehicles for career readiness remains underexplored, particularly within online delivery contexts. This gap in practice signals an opportunity to reconceptualize advising not as a fixed set of interactions, but as a continuum, one that creates multiple pathways for students: toward degree completion, toward personal and professional growth, and toward the career confidence and self-efficacy they need to enter the public health workforce prepared to lead.

## 3 Updated advising approach

### 3.1 Advising continuum—a framework to impact students professional readiness efficacy

**Figure 1:**
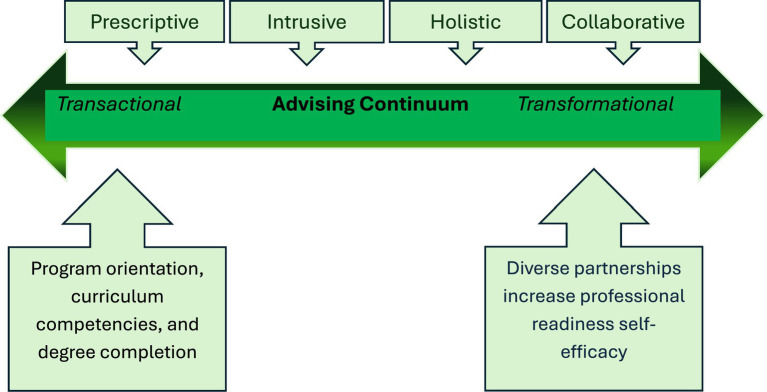
Michigan State University Master of Public Health’s Advising Continuum. *Adapted from: Collaboration: A School Improvement Strategy (23).

Academic advising has traditionally been viewed as a relationship centered on academic planning, course selection, and degree completion. While these functions remain essential, student success efforts require a multifaceted approach that supports students’ academic, personal, and professional development. This advising continuum (Figure 1) builds upon traditional advising practices while introducing a more intentional, integrated approach to help develop professional readiness self-efficacy among public health students. It consists of four connected orientations: prescriptive, intrusive, holistic, and collaborative advising; that can be implemented independently or in combination to address students’ evolving needs.

Starting from the transactional side of this continuum, prescriptive advising focuses on academic planning, degree requirements, and curricular progression, while intrusive advising employs proactive outreach and targeted interventions to address barriers that may limit student success. Then, holistic advising extends beyond academic concerns to support the whole student by recognizing how personal, social, and professional factors shape student success. This includes attentiveness to student mental health and well-being, an increasingly critical dimension of graduate student support, with research suggesting approximately 40% of graduate students experience significant mental health challenges such as anxiety, depression, or burnout (21). Advisors operating within the holistic orientation are positioned to notice early signs of distress and connect students to appropriate institutional resources, even as the advising role itself remains distinct from clinical mental health support.

At the most transformative end of the continuum is collaborative advising, a relational and student-centered orientation that expand the advisor’s role beyond the traditional student-advisor relationship to include intentional partnerships with faculty, alumni, employers, mentors, and community organizations. Through these collaborative relationships, students are exposed to professional networks, experiential learning opportunities, workforce pathways, and mentorship that support academic development, professional socialization, and career readiness. Collaborative advising integrates both transactional and transformational practices by aligning competency-based learning with real-world applications and helping students to develop their professional self-efficacy. We contend that this collaborative orientation represents a meaningful advising response that positions advising not merely as an academic support service, but as a mechanism for preparing students to transition into the public health workforce and thrive as confident professionals.

### 3.2 Advising continuum in practice

Within this MPH program, collaborative advising is now operationalized through an approach that relies on internal, external, and cross-departmental partnerships. The advisors implementing this continuum are academic advising staff, not teaching or research faculty, who hold student support as their primary institutional role within the online MPH program. Advising extends beyond traditional course planning through regular, intentional meetings that integrate these elements and pathways into public health practice. Advisors serve as connectors, linking students to internal and external opportunities formed by these partnerships.

As an example of internal partnerships, working with faculty mentorship further strengthens this model through coordinated engagement between advisors and mentors, creating a wraparound approach that supports both academic progression and professional development. Internal and external partnerships expand career readiness pathways by connecting students to public health organizations, community-based experiences, cross-departmental collaborations, and graduate certificate opportunities that strengthen interdisciplinary learning and support both current professionals and students transitioning into public health.

The program also incorporates practical career development strategies, including weekly dissemination of job opportunities and resources, resume and CV development checkpoints, and networking and professional development guidance. Collectively, these efforts position collaborative advising as a proactive and relational approach that extends beyond traditional advising strengthening professional identity formation, and readiness to enter the workforce.

## 4 Findings

### 4.1 Methods

End-of-semester student advising perceptions survey was administered via Qualtrics between academic years 2023 through 2026 captured perceptions of advising quality, professional readiness support, and the overall effectiveness. The survey included Likert-scale items assessing preparedness, responsiveness, and overall satisfaction, as well as targeted questions about the presence and quality of career advising conversations. Student responses are reviewed each semester to identify areas of strength and opportunities to deepen this collaborative approach.

Data drawn from this survey across three academic years provide meaningful evidence of the advising continuum approach’s growing impact on student experience and professional readiness. Table 1 presents findings across 2023–24, 2024–25, and 2025–26, illustrating an upward trajectory across all three measured dimensions as the continuum has been more intentionally implemented over time.

**Table 1:** Summary of student advising findings (Spring 2023–Spring 2026).

Key metric	23–24 academic year (*n* = 23)	24–25 academic year (*n* = 32)	25–26 academic year (*n* = 11)	Emerging themes
Overall advising satisfaction	Rated experience as positive	Rated experience as positive	Rated experience as positive	
	39.1%	84.4%	100.0%	Transactional builds student confidence
Career advising discussions with advisor	Reported having career advising conversations	Reported having career advising conversations	Reported having career advising conversations	
	21.7%	25.0%	36.4%	Transformational collaborative touchpoints expand professional awareness
Advisor meeting career advising needs	Felt advisor was meeting career advising needs	Felt advisor was meeting career advising needs	Felt advisor was meeting career advising needs	
	47.8%	78.1%	100.0%	Transformational collaborative touchpoints are associated with professional identity growth.

Only fully completed survey responses were included. AY 2025–26 reflects data collected through May 2026 and does not represent a complete academic year.

### 4.2 Results

Overall, advising satisfaction rose from 39.1% in AY 2023–24 to 84.4% in AY 2024–25 and 100.0% in AY 2025–26. Similarly, the percentage of students reporting their career advising needs were met climbed from 47.8 to 78.1 to 100.0% across the same period. Career advising discussions, while still developing in practice, also showed steady growth from 21.7 to 25.0 to 36.4% reflecting incremental but meaningful progress in expanding the reach of collaborative advising touchpoints. It is worth noting that while AY 2025–26 data are not yet complete, the 100.0% satisfaction and career advising needs met figures across all respondents to date are a promising indicator of sustained growth and a meaningful marker of the continuum’s impact even in the absence of a full year of data. Across all three academic years, students consistently described advisors who were prepared, proactive, and personally invested in their success, noting that structured meetings, timely follow-up communications, and a genuine sense of being known were among the most valuable aspects of their experience. Advising has expanded the topics that are brought in communications like post-graduation plans and started new practices like sending weekly job postings. These relational qualities preparation, responsiveness, and personalized engagement are the hallmarks of the advising continuum approach and appear to be directly associated with students’ increased comfort navigating the program and their growing confidence in their professional pathways.

Three themes emerged consistently across the data as markers of the collaborative advising approach’s impact. Students who experienced proactive, transformational touchpoints reported greater confidence and a clearer sense of their professional pathway readiness. Collaborative tools, career planning documents, job postings, and others were identified as meaningful touchpoints that expanded professional awareness beyond the classroom. Importantly, they distinguished between advising that felt transactional and advising that felt transformational, and it was the transformational side they associated with professional identity growth and readiness.

## 5 Discussion

### 5.1 Advising as a continuum can help students stay on the pathway to graduation

Advising as a continuum enables advisors to meet students where they are while systematically cultivating the knowledge, skills, and networks necessary for their success as public health professionals. It also has practical implications for MPH programs seeking to strengthen retention, completion, and professional readiness. We have adopted flexible advising strategies to engage prescriptive, intrusive, holistic, and collaborative strategies because we know the online MPH students routinely navigate overlapping responsibilities and commitments. A continuum allows us to recognize that, as advisors, we need to engage with students using a spectrum from directive to facilitative, informed by student readiness, life context, and workplace needs. This continuum is flexible and iterative: advisors may rapidly shift from prescriptive course planning to intrusive engagement to ensure students’ ability to progress on the degree completion pathway. By employing responsiveness and informed professional judgment, the continuum supports individualized engagement that consider students’ needs. Our survey data affirms this: students who experienced proactive, transformational advising reported greater confidence, strong professional identify, and a clearer sense of their pathway into the public health workforce, outcomes that reflect not just academic progress, but the growth in professional self-efficacy that transactional advising alone cannot produce.

Effective advising integrates these orientations: for example, a prescriptive semester plan can be paired with holistic career mapping and early collaborative connections and community-based opportunities. Advisors should be ready to move along the continuum, using transactional touchpoints as sites for identifying candidates for transformational engagement. Such intentional sequencing enhances professional readiness by ensuring that students not only complete requirements but also practice competencies and build professional networks and develop self-efficacy to navigate their careers with confidence.

An important and often underacknowledged dimension of the advising continuum is its role in student mental health and well-being. Research suggests that a growing number of graduate students experience significant mental health challenges that can directly impede academic progress and professional development (21, 22). Within our continuum, the holistic advising orientation is particularly positioned to attend to these needs. By maintaining regular, relational touchpoints and genuinely knowing their students, advisors are often among the first to notice when a student may be struggling. While advisors are not mental health clinicians, the continuum’s emphasis on proactive outreach and individualized engagement positions them to offer a supportive presence and make timely referrals to counseling, disability services, or other wellness resources. Future iterations of this work may benefit from more explicit protocols for mental health-informed advising, including structured check-ins and advisor training in recognizing early indicators of distress.

### 5.2 Collaborative advising: a transformational pathway

What our continuum suggests, and what we did not find well-articulated in the literature, is that collaborative advising represents a distinct and necessary orientation beyond holistic advising. Where holistic advising attends to the whole student within the advising relationship, collaborative advising extends outward: it positions the advisor as a connector, bridgebuilder, and active architect of the student’s professional future. For online MPH students who lack the informal networking that in-person programs naturally provide, this outward reach is not supplemental but essential. Our findings support this: students who engaged with collaborative touchpoints like career planning documents, professional job dissemination, mentorship connections, and community partnerships described a qualitatively different advising experience, one that moved them from feeling supported academically to feeling prepared professionally and confident in their pathways into the public health workforce. This distinction, between being known by your advisor and being connected by your advisor, is at the heart of what the collaborative orientation adds to the continuum.

## 6 Limitations and future directions

This work is not without limitations. Data was drawn from a single online MPH program, and findings reflect one program’s experience rather than a field-wide pattern. The modest percentages of students reporting career advising conversations and mentor engagement also suggest that the collaborative orientation, while promising, is still developing in practice and has not yet reached all students consistently. Additionally, this data drew exclusively on student perceptions of advising quality and professional readiness and does not capture advisor perception of the continuum or its implementation; future research examining how advisors experience and navigate the continuum, including the institutional conditions that enable or constrain their practice, would provide a valuable complementary perspective. Future work should include greater levels of response and examine how the advising continuum framework translates across program types, institutional contexts, and student populations, particularly programs serving different demographic profiles or operating under different accreditation structures. We also recognize that fully enacting collaborative advising requires institutional investment in advisor capacity, partnership development, and structured mentorship infrastructure. Nevertheless, the evidence presented here suggests that when advisors move deliberately along the continuum, from transactional to transformational, students notice. Programs seeking to strengthen professional readiness do not need to overhaul their curricula; they may start by reconsidering what partnerships they can build.

## Data Availability

The original contributions presented in the study are included in the article/Supplementary material, further inquiries can be directed to the corresponding author.
